# Neuronal Adenosine A_1_ Receptor is Critical for Olfactory Function but Unable to Attenuate Olfactory Dysfunction in Neuroinflammation

**DOI:** 10.3389/fncel.2022.912030

**Published:** 2022-06-30

**Authors:** Charlotte Schubert, Kristina Schulz, Simone Träger, Anna-Lena Plath, Asina Omriouate, Sina C. Rosenkranz, Fabio Morellini, Manuel A. Friese, Daniela Hirnet

**Affiliations:** ^1^Institute of Neuroimmunology and Multiple Sclerosis (INIMS), University Medical Center Hamburg-Eppendorf, Hamburg, Germany; ^2^Division of Neurophysiology, Institute of Cell and Systems Biology of Animals, University of Hamburg, Hamburg, Germany; ^3^Research Group Behavioral Biology, Center for Molecular Neurobiology (ZMNH), University Medical Center Hamburg-Eppendorf, Hamburg, Germany

**Keywords:** adenosine, A_1_R, purinergic signaling, EAE, olfactory bulb, neuroprotection, mitral cells, olfactory dysfunction

## Abstract

Adenine nucleotides, such as adenosine triphosphate (ATP), adenosine diphosphate (ADP), as well as the nucleoside adenosine are important modulators of neuronal function by engaging P1 and P2 purinergic receptors. In mitral cells, signaling of the G protein-coupled P1 receptor adenosine 1 receptor (A_1_R) affects the olfactory sensory pathway by regulating high voltage-activated calcium channels and two-pore domain potassium (K2P) channels. The inflammation of the central nervous system (CNS) impairs the olfactory function and gives rise to large amounts of extracellular ATP and adenosine, which act as pro-inflammatory and anti-inflammatory mediators, respectively. However, it is unclear whether neuronal A_1_R in the olfactory bulb modulates the sensory function and how this is impacted by inflammation. Here, we show that signaling *via* neuronal A_1_R is important for the physiological olfactory function, while it cannot counteract inflammation-induced hyperexcitability and olfactory deficit. Using neuron-specific A_1_R-deficient mice in patch-clamp recordings, we found that adenosine modulates spontaneous dendro-dendritic signaling in mitral and granule cells *via* A_1_R. Furthermore, neuronal A_1_R deficiency resulted in olfactory dysfunction in two separate olfactory tests. In mice with experimental autoimmune encephalomyelitis (EAE), we detected immune cell infiltration and microglia activation in the olfactory bulb as well as hyperexcitability of mitral cells and olfactory dysfunction. However, neuron-specific A_1_R activity was unable to attenuate glutamate excitotoxicity in the primary olfactory bulb neurons *in vitro* or EAE-induced olfactory dysfunction and disease severity *in vivo*. Together, we demonstrate that A_1_R modulates the dendro-dendritic inhibition (DDI) at the site of mitral and granule cells and impacts the processing of the olfactory sensory information, while A_1_R activity was unable to counteract inflammation-induced hyperexcitability.

## Introduction

Adenine nucleotides are essential neurotransmitters in cellular homeostasis and disease. During synaptic transmission and also under inflammatory conditions, neurons and astrocytes can release adenosine triphosphate (ATP) (Burnstock et al., 2011). This is then rapidly degraded to adenosine diphosphate (ADP) and subsequently processed to adenosine monophosphate (AMP). and adenosine by the ectoenzymes CD39 (ectonucleoside triphosphate diphosphohydrolase-1) and CD73 (5′-ribonucleotide phosphohydrolase) (Dunwiddie et al., 1997; Sebastiao et al., 1999; Zimmermann, 2021). Extracellular adenosine binds and promotes its main cellular functions *via* the purinergic P1 receptors A_1_, A_2A_, A_2B_, and A_3_ (Fredholm et al., 2011). In the central nervous system (CNS), ample evidence suggests that the functions of adenosine are predominantly mediated by the high-affinity A_1_R (Fredholm et al., 2001) as well as the A_2a_R (Gomes et al., 2011; Stockwell et al., 2017). A_1_R is mainly coupled to the pertussis toxin (PTX)-sensitive G proteins of the G_α__*i*_ and G_α__*o*_ families of the guanine nucleotide-binding proteins (Munshi et al., 1991). Consequently, neuronal stimulation of A_1_R leads to the inhibition of adenylyl cyclase (AC) and reduction of cAMP production, as well as the inhibition of the cAMP-dependent kinase (PKA), which modulates the release of neurotransmitters and neuropeptides (Carruthers et al., 2001; Jeong et al., 2003). Other targets of G_α__*i*_ include K^+^ channels (Kirsch et al., 1990; Sickmann and Alzheimer, 2003; Kim and Johnston, 2015) and Ca^2+^ channels in the CNS (Liu and Gao, 2007). Of note, stimulation of A_1_R also activates the phospholipase C (PLC) pathway *via* the release of G_β__γ_ dimers in various cell types (Biber et al., 1997; Dickenson et al., 1998; Fenton et al., 2010).

Neuromodulation by adenosine is of critical importance to a wide variety of CNS functions (Gourine et al., 2009; Nazario et al., 2017; Ballesteros-Yáñez et al., 2018; Lazarus et al., 2019; Jagannath et al., 2021) and recently we also found that it is involved in olfactory information processing (Rotermund et al., 2018; Schulz et al., 2018). Olfactory signaling is provided by an interplay of several cell types. After odor detection at the site of the olfactory sensory neurons, the signal converges on glomerular columns and is processed by several inhibitory neurons, such as periglomerular interneurons and granule cells (Egger and Urban, 2006; Wachowiak and Shipley, 2006; Linster and Cleland, 2009; Burton, 2017). The projection neurons, mitral, and tufted cells integrate all inputs to create the output signal of the olfactory bulb (Geramita et al., 2016) and transmit it to higher cortical areas of the olfactory pathway, such as the anterior olfactory nucleus and the piriform and entorhinal cortices (Neville and Haberly, 2004; Isaacson, 2010; Rotermund et al., 2019). Dendrites of the mitral cells form specialized reciprocal synapses with dendrites of the glomerular interneurons and granule cells that result in a mechanism termed dendro-dendritic inhibition (DDI) (Rall et al., 1966; Isaacson and Strowbridge, 1998; Schoppa, 2006; Balu et al., 2007; Todd Pressler and Strowbridge, 2020). In the main olfactory bulb A_1_R and to a less extent A_2A_R are expressed in mitral cells (Reppert et al., 1991; Lein et al., 2006; Rotermund et al., 2018). Activation of A_1_R on mitral cells hyperpolarizes the cell by increasing the potassium currents through two-pore domain potassium (K2P) channels. This hyperpolarization decreases firing frequency of mitral cells at rest but has no impact on the firing frequency upon stimulation of sensory axons mimicking sensory input into the cell. Thereby, adenosine might increase the signal-to-noise-ratio detecting olfactory stimuli (Rotermund et al., 2018). In addition, adenosine inhibits L- and P/Q-type calcium channels by reducing the calcium-dependent vesicular glutamate release of mitral cells. At reciprocal synapses with GABAergic granule cells and parvalbumin interneurons, the reduced glutamate release in turn decreases DDI (Schulz et al., 2018).

During chronic autoimmune CNS inflammation, as can be detected during multiple sclerosis (MS), the olfactory function is compromised (Lucassen et al., 2016) and correlates with disease duration and severity (Yang et al., 2016; Uecker et al., 2017; Bsteh et al., 2019). Of note, olfactory dysfunction is already detectable in patients early at the onset of the disease, where the patients experience increased olfactory threshold in four out of five cases (Lutterotti et al., 2011). In primary-progressive MS (PPMS), around 84% of patients suffer from olfactory dysfunction affecting the olfactory threshold and odor identification and discrimination (Joseph and De Luca, 2016).

Disease severity in inflammatory diseases, such as MS and its animal model EAE are modulated by the extent of immune cell infiltration, immune cell composition, and their effector function (Attfield et al., 2022). Besides neuron- and glial-intrinsic properties play a major role in promoting or inhibiting neurodegeneration (Friese et al., 2014; Healy et al., 2022; Lee et al., 2022). This is particularly relevant in the progressive disease stages of MS, mirrored by the chronic phase of the EAE. In neuroinflammation glutamate, excitotoxicity is one of the main detrimental causes resulting in neurodegeneration (Woo et al., 2021). In inflammation, ATP is released into the extracellular space, where it exerts pro-inflammatory functions (Cauwels et al., 2014; Jesudasan et al., 2021). On the other hand, adenosine, as a degradation product of the enzymatic conversion of ATP by CD39 and CD73, promotes an anti-inflammatory milieu (Schetinger et al., 2007; Deaglio and Robson, 2011). Moreover, several studies indicate neuroprotective effects by adenosine in the CNS (Chen et al., 2001; Cunha, 2016; Coppi et al., 2021). However, the relative contribution of ATP- and adenosine-dependent neuronal signaling during neuroinflammation in driving or protecting against olfactory injury is unknown. Adenosine could potentially suppress neuronal excitability by restricting calcium influx and inhibiting glutamate release, eventually counteracting neuronal calcium overload (Corradetti et al., 1984; Dunwiddie et al., 1984; Pereira-Figueiredo et al., 2021). This could be mediated presynaptically, as A_1_ activation inhibits voltage-activated calcium channels (Ca_*v*_) reducing transmitter exocytosis (Banie and Nicholls, 1993; Zhu and Ikeda, 1993; Umemiya and Berger, 1994; Gundlfinger et al., 2007). In addition, the postsynaptic adenosine-induced opening of potassium channels and the consecutive hyperpolarization prevents the activation of Ca_*v*_ and stabilizes the Mg^2+^ block of NMDA receptor channels, thereby avoiding excessive neuronal calcium influx (Trussell and Jackson, 1985; de Mendonça et al., 1995). Accordingly, ischemia-induced synaptic depression is substantially reduced in the hippocampal slices of A_1_-deficient mice (Johansson et al., 2001; Kawamura et al., 2019). Similarly, A_1_-deficient mice show an ameliorated disease course of experimental autoimmune encephalomyelitis (EAE) (Tsutsui et al., 2004), the animal model of MS. However, these EAE experiments used constitutive A_1_-deficient mice of the entire organism and did not allow to distinguish A_1_-dependent functions of immune cells from those of inflamed neurons. Here, we set out to disentangle the role of neuronal A_1_R purinergic signaling in the olfactory bulb under healthy and inflammatory conditions. In healthy conditions, patch clamp recordings of olfactory bulb mitral and granule cells showed A_1_R-dependent modulation of dendro-dendritic signaling, and a lack of A_1_R expression in neurons impaired the performance of the animals in olfactory behavior tests. During neuroinflammation, the gain of the current–frequency relationship of mitral cells increased, yet A_1_R deficiency neither modulated this rise in gain nor the extent of neurodegeneration or impaired the olfactory performance.

## Results

### Neuronal Adenosine 1 Receptor in Homeostatic Conditions

**FIGURE 1:**
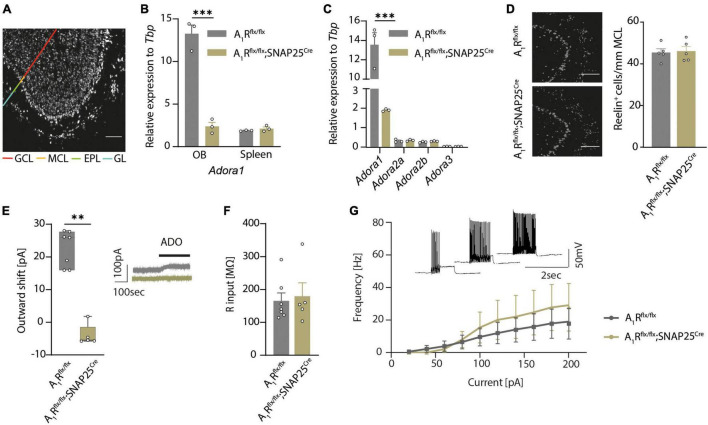
A_1_R*^flx/flx^*;SNAP25*^Cre^* results in neuron-specific A1R knockout. **(A)** HA-tagged reporter expression in neuron-specific A_1_R knockout mouse line A_1_R*^flx/flx^*;Tag;SNAP25*^Cre^* indicates wide distribution of Cre expression in the main olfactory bulb. Representative image shows a sagittal view of the main olfactory bulb comprising the granular cell layer (GCL), internal plexiform layer (not marked), mitral cell layer (MCL), external plexiform layer (EPL), and the glomerular layer (GL) as indicated by different colors. HA-tag was stained with anti-HA antibody. **(B)** Quantitative qPCR of *Adora1* (coding for the A_1_R) mRNA expression of olfactory bulbs and spleens (BF_10_ = 0.538) isolated from A_1_R*^flx/flx^* (*n* = 3) and A_1_R*^flx/flx^*;SNAP25*^Cre^* (*n* = 3). **(C)** Quantitative qPCR mRNA expression of P1 receptor genes *Adora1*, *Adora2a* (BF_10_ = 0.646), *Adora2b* (BF_10_ = 0.624), and *Adora3* (BF_10_ = 0.565) in the olfactory bulb of A_1_R*^flx/flx^* (*n* = 3) and A_1_R*^flx/flx^*;SNAP25*^Cre^* (*n* = 3) mice. **(D)** Mitral cells analyzed by marker Reelin in the mitral cell layer in A_1_R*^flx/flx^* (*n* = 5) and A_1_R*^flx/flx^*;SNAP25*^Cre^* (*n* = 5) mice (BF_10_ = 0.337). **(E)** Outward shift of the holding current in the mitral cell patch clamp recordings evoked by the application of 100 μM of adenosine (ADO) in A_1_R*^flx/flx^* (*n* = 7 slices from 5 mice) and A_1_R*^flx/flx^*;SNAP25*^Cre^* (*n* = 5 slices from 4 mice) (MWU; *U* = 0, *P* = 0.0025). Representative traces in gray (A_1_R*^flx/flx^*) and olive (A_1_R*^flx/flx^*;SNAP25*^Cre^*). **(F)** Input resistance was measured in patch clamp recordings of mitral cells from A_1_R*^flx/flx^* (*n* = 7 slices from 5 mice) and A_1_R*^flx/flx^*SNAP25*^Cre^* (*n* = 5 slices from 4 mice) mice, BF_10_ = 0.486. **(G)** Progression of the F/I plot shows excitability of mitral cells from A_1_R*^flx/flx^* (*n* = 7 slices from 5 mice) mice vs. A_1_R*^flx/flx^*;SNAP25*^Cre^* (*n* = 5 slices from 4 mice) (BF_10_ = 0.480-0.537). Data points display mean ± s.e.m. ^**^P < 0.01, ^***^P < 0.001.

To disentangle the olfactory role of the neuronal A_1_ receptor in health and neuroinflammation, we first generated a neuron-specific A_1_R-deficient mouse line. We bred mice with a floxed *Adora1* gene, which encodes for A_1_R (Scammell et al., 2003), with mice that express the Cre recombinase driven by the SNAP25 promoter (Zitman and Richter-Levin, 2013). By further breeding the A_1_R*^flx/flx^*;SNAP25*^Cre^* mice to *tagger* mice (Tag) that contain a Cre-dependent nuclear fluorescent tag as well as an HA tag (Kaczmarczyk et al., 2019), we could visualize neuron-specific expression in almost all brain regions, including the olfactory bulb (Figure 1A), cortex, hippocampus, and the spinal cord (Supplementary Figures 1A–C). Validation of reporter expression in specific neuronal subsets in the olfactory bulb revealed labeling of approximately 80% of NeuN-positive neurons in the granular cell layer and slightly fewer in the glomerular layer of the main olfactory bulb (data not shown) and 87% of the reelin-positive mitral cells in the mitral cell layer (Supplementary Figure 1D). Specific labeling of neurons using the SNAP25*^Cre^* mouse was confirmed by the staining of glial markers Iba1 (microglia), GFAP (astrocytes), and CNPase (oligodendrocytes) (Supplementary Figures 1E–G). While *Adora1* expression was unaltered in the spleen in qPCR (Figure 1B), *Adora1* expression was significantly reduced in the olfactory bulb tissue of A_1_R*^flx/flx^*;SNAP25*^Cre^* suggesting a neuron-specific knockout (*n* = 3 per group, Figure 1B). In addition, we confirmed a reduction of A_1_R in primary cell cultures by immunocytochemistry (*n* = 8 per group in *n* = 4 biological replicates, Supplementary Figure 1H). In contrast, mRNA expression levels of the P1 receptors A_2a_ (*Adora2a*), A_2b_ (*Adora2b*), and A_3_ (*Adora3*) did not differ in the olfactory bulb tissue of A_1_R-deficient mice compared to their wildtype littermates, suggesting no relevant compensatory mechanisms in the transcriptional levels in these mice (*n* = 3 per group, Figure 1C).

By comparing A_1_R*^flx/flx^* and A_1_R*^flx/flx^*;SNAP25*^Cre^* we did not observe apparent phenotypic alterations, including body weight (Supplementary Figure 1I). However, neuron-specific A_1_R deficiency resulted in slightly reduced survival rate of A_1_R*^flx/flx^*;SNAP25*^Cre^* mice compared to their floxed littermates. At 15 weeks of age, 7% of A_1_R*^flx/flx^*;SNAP25*^Cre^* mice showed unexpected death while only 3% of A_1_R*^flx/flx^* mice (Supplementary Figure 1J) died. Moreover, neuronal cell count in the olfactory bulbs of wildtype and A_1_R knockout mice showed no differences in mitral cell counts, (Figure 1D), suggesting no overt developmental differences. Thus, having a validated neuron-specific A_1_R-deficient mouse line enabled us to disentangle the role of the neuronal A_1_R function in health and in neuroinflammation.

Since we previously showed that activation of A_1_R in mitral cells led to an increase of background potassium conductance by two-pore-domain potassium channel subfamily (K2P) (Rotermund et al., 2018), we interrogated whether A_1_R*^flx/flx^*;SNAP25*^Cre^* shows a functional abrogation in whole-cell recordings of mitral cells in olfactory bulb slices. Application of adenosine induced an outward shift of the holding current in A_1_R*^flx/flx^* animals (23 ± 2 pA, *n* = 7) but failed to increase the background current in the mitral cells of A_1_R*^flx/flx^*;SNAP25*^Cre^* mice (*n* = 5) (Figure 1E). Next, we examined whether the lack of A_1_R in mitral cells has an impact on neuronal properties of mitral cells. Consistent with the data of global A_1_R knockout mice (Johansson et al., 2001), we did not find a significant difference in the resting membrane potential (−41.4 ± 0.8 mV in A_1_R*^flx/flx^* vs. −44.5 ± 2.1 mV in A_1_R*^flx/flx^*;SNAP25*^Cre^*) (Supplementary Figure 2A) or input resistance (165 ± 23 MΩ in A_1_R*^flx/flx^* vs. 179 ± 40 MΩ in A_1_R*^flx/flx^*;SNAP25*^Cre^*) of the recorded cells (Figure 1F). Stepwise increase of the commando potential and the recording of the corresponding whole-cell conductance to illustrate the current–voltage relationship also did not reveal any differences between A_1_R-proficient and A_1_R-deficient mitral cells (Supplementary Figure 2B). To analyze potential differences in firing behavior, we applied depolarizing current injections of consecutively higher amplitude and plotted the frequency of the induced action potentials of mitral cells. Neither the rheobase, the first current step resulting in a membrane depolarization beyond the action potential threshold (Supplementary Figure 2C), nor the progression of the F/I plot showed significant alterations in the excitability of A_1_R*^flx/flx^*;SNAP25*^Cre^* mitral cells in comparison to the A_1_R-proficient cells (Figure 1G). Hence, while membrane properties of mitral cells in A_1_R*^flx/flx^*;SNAP25*^Cre^* seem unaltered, mitral cells show a profound deficit in response to extracellular adenosine concentrations with K2P activation.

### Neuronal Adenosine 1 Receptor is Involved in Olfactory Dendro-Dendritic Transmission

**FIGURE 2:**
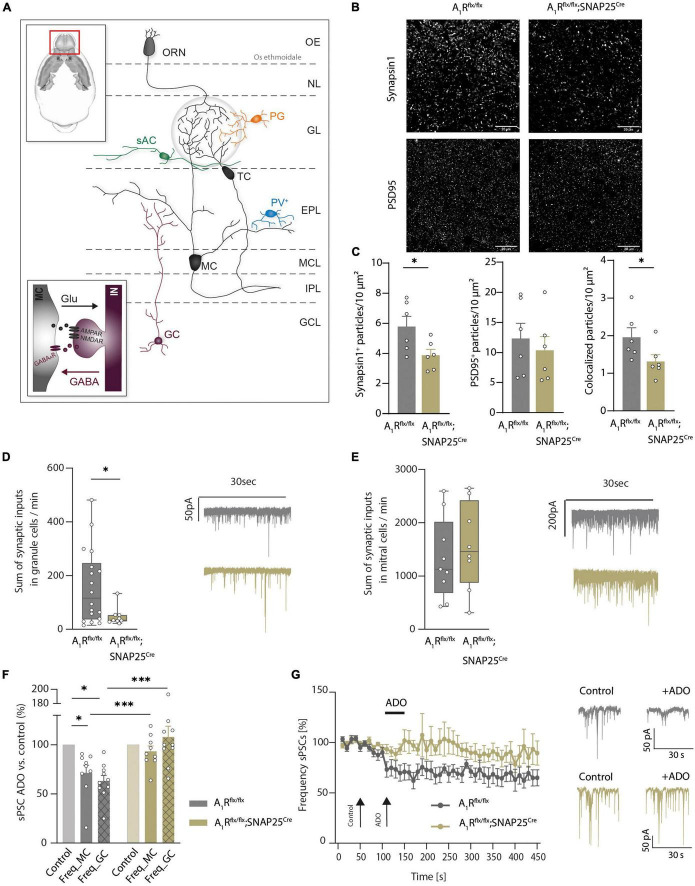
Adenosine modulates microcircuits in the external plexiform layer of the main olfactory bulb *via* A_1_R. **(A)** Schematic presentation of main olfactory bulb with principal neurons (MC, TC) and local interneurons [periglomerular neurons (PG), short axon cells (sAC), parvalbumin neurons (PV), and granule cells (GC)]. Inset: Mechanism of dendro-dendritic signaling at the reciprocal MC-GC synapse. **(B)** Representative images of synaptic density in the external plexiform layer (EPL) of the main olfactory bulb in A_1_R-proficient (A_1_R*^flx/flx^*) and A_1_R-deficient (A_1_R*^flx/flx^*;SNAP25*^Cre^*) mice. Immunohistochemical stainings comprised the presynaptic protein synapsin1 and the postsynaptic protein PSD95. **(C)** Immunohistochemical analysis of synapsin1, PSD95 (BF_10_ = 0.518), and co-expression of synapsin1 and PSD95 in the EPL in A_1_R*^flx/flx^* and A_1_R*^flx/flx^*;SNAP25*^Cre^* (*n* = 6 per group). **(D)** Whole cell current recordings of granule cells. Number of synaptic inputs quantified as the sum of sPSC events per minute in A_1_R*^flx/flx^* (*n* = 18) compared to A_1_R*^flx/flx^*;SNAP25*^Cre^* (*n* = 9). MWU; *U* = 40, *P* = 0.035. **(E)** Whole cell current recordings of mitral cells. Number of synaptic inputs quantified as the sum of sPSC events per minute (A_1_R*^flx/flx^ n* = 9; A_1_R*^flx/flx^*;SNAP25*^Cre^ n* = 8), BF_10_ = 0.476. **(F)** Frequency of sPSCs in mitral cells and granule cells after the application of 100 μM of adenosine (mean of second 130–170 of recording) compared to baseline (mean of second 30–70 of recording) in A_1_R*^flx/flx^* (*n* = 10 in granule cells, *n* = 9 in mitral cells) and A_1_R*^flx/flx^*;SNAP25*^Cre^* (*n* = 10 in granule cells, *n* = 9 in mitral cells). Adenosine-dependent effect on sPSC frequency was analyzed by the Wilcoxon ranking test. For A1R-proficient cells, *P* = 0.00195 (granule cells) and *P* = 0.00391 (mitral cells); for A1R-deficient cells, BF_10_ (paired test) = 0.322 (granule cells) and BF_10_ (paired test) = 0.793 (mitral cells). Genotype comparison was done by Mann–Whitney U test. Adenosine effect A_1_R*^flx/flx^* vs. A_1_R*^flx/flx^*;SNAP25*^Cre^* for granule cells (*U* = 6, *P* = 0.003) and mitral cells (*U* = 15, *P* = 0.024). **(G)** Frequency of sPSCs in mitral cells normalized to baseline over the time course of >7 min. Representative traces showing sPSC of mitral cells under baseline condition and after treatment with 100 μM of adenosine at timespoints indicated by arrows. *P < 0.05, ^***^P < 0.001.

Having shown the A_1_R-dependent modulation of presynaptic calcium channels in mitral cells (Schulz et al., 2018), we next investigated the structural and functional role of A_1_R on synaptic connections of mitral cells in the external plexiform layer. Here mitral cells are forming dendro-dendritic synapses with several GABAergic interneurons, mainly granular cells. These reciprocal connections are involved in recurrent and lateral inhibition of excited mitral cells by granule cells (Isaacson and Strowbridge, 1998; Egger and Kuner, 2021). Schematic representation is shown in Figure 2A. Neuronal A_1_R deficiency resulted in a reduced density of glutamatergic synapses as recorded by synapsin1 and PSD-95 expression and colocalization (*n* = 6 per group, Figures 2B,C). Yet, inhibitory synapses in the external plexiform layer were not significantly affected (Supplementary Figure 2D). As this reciprocal connection of mitral cells with modulating granular cells seems to be affected by the lack of the A_1_R, we further investigated the synaptic functions by patch-clamp recordings.

By recording spontaneous inhibitory and excitatory synaptic currents (sPSCs) in granule cells using high chloride intracellular solution, we revealed a lower frequency of inputs in A_1_R*^flx/flx^*;SNAP25*^Cre^* animals compared to A_1_R*^flx/flx^* controls (47.61 ± 11.37, *n* = 10, vs. 158.19 ± 32.61, *n* = 10, mean summarized number of inputs per minute, Figure 2D). In contrast, recording sPSCs in mitral cells revealed no differences in the baseline activity (1326 ± 253 in A_1_R*^flx/flx^*, *n* = 9, vs. 1559 ± 291 in A_1_R*^flx/flx^*;SNAP25*^Cre^*, *n* = 8, mean summarized number of inputs per minute) in the recorded sample (Figure 2E). Application of 100 μM of adenosine significantly reduced sPSC frequency in the granule cells of A_1_R*^flx/flx^* animals by 36.91 ± 5.74% (*n* = 10), whereas in the granule cells of A_1_R*^flx/flx^*;SNAP25*^Cre^* sPSC frequency did not change upon adenosine application compared to baseline values (frequency elevated to 7.9 ± 11%, *n* = 10), confirming the absence of A_1_R-induced effects in the A_1_R knockout. Concomitantly, wildtype mitral cells showed a significant reduction of synaptic input frequency upon the application of 100 μM of adenosine (28.6 ± 7.8%, *n* = 9) but not the mitral cells of A_1_R*^flx/flx^* SNAP25*^Cre^* animals (frequency lowered by 6.7 ± 5.4%, *n* = 9) (Figure 2F). Time course analysis confirmed the reduction of synaptic input frequency by adenosine in A_1_R-proficient, but not in A_1_R-deficient mitral cells over at least 6 min (Figure 2G). Taken together, these data corroborate the A_1_R-dependent modulation of dendro-dendritic circuits by adenosine.

### Neuronal Adenosine 1 Receptor is Critical for Olfactory Function

**FIGURE 3:**
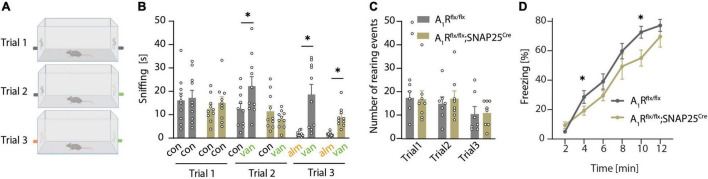
A_1_R deficiency results in olfactory dysfunction. **(A)** Graphical workflow of olfactory detection test. A_1_R*^flx/flx^* and A_1_R*^flx/flx^*;SNAP25*^Cre^* were exposed to empty tubes (black), or tubes perfumed with a concentrate of vanilla (green) or almond (orange). Each trial lasted 4 min. **(B)** Quantification of olfactory detection tests. Preference to new odor analyzed by the time of sniffing at the respective side (s) (*n* = 10 per group, BF_10_ = 0.361). **(C)** Events of rearing during each trial in olfactory detection test of A_1_R*^flx/flx^* and A_1_R*^flx/flx^*;SNAP25*^Cre^* mice (BF_10_ = 0.439–0.473). **(D)** TMT-based olfactory test. Time of freezing in percent in bins of 2 min in A_1_R*^flx/flx^* (*n* = 11) and A_1_R*^flx/flx^*;SNAP25*^Cre^* (*n* = 6). Bonferroni *post-hoc* test after a mixed multifactorial ANOVA; *P < 0.05.

As A_1_R is important for the DDI of mitral cells, we next explored whether that translates to an olfaction phenotype using a two-sided olfactory detection test with the odor of vanilla and almond (Figure 3A). During the first trial (when no odor was presented), mice of both genotypes (*n* = 10 per group) spent the same amount of time sniffing at the two openings at the left and right sides of the cage (Figure 3B) and did not show differences in rearing events, which reflects a similar exploratory behavior (Figure 3C). Thus, neuronal A_1_R deletion did not affect the general exploratory and sniffing behavior, and there was no bias toward one side of the cage. In trial 2, an unfamiliar odor (vanilla) was presented at the right side of the cage (Figure 3B). Whereas A_1_R*^flx/flx^* control mice preferentially sniffed at the opening with vanilla, A_1_R*^flx/flx^*;SNAP25*^Cre^* did not display a preference for the new odor (Figure 3B). In trial 3, we introduced the odor almond at the side opposite vanilla (Figure 3A). We used almond as a new odor with the expectation that mice would investigate it more intensely than the already familiar vanilla. However, all mice spent only few seconds sniffing it, indicating that almond flavor induced an aversive reaction, at least under these experimental conditions (Figure 3B). Whereas these results suggest that neuronal A_1_R deficiency impairs olfaction; we cannot exclude that an altered novelty-induced behavior of the A_1_R*^flx/flx^*;SNAP25*^Cre^* mice caused a lack of preference for the new odor of vanilla. We thus tested olfaction in a task that relies on an innate behavior to 2,5-dihydro-2,4,5-trimethylthiazoline (TMT), a constituent of fox urine and feces (Saito et al., 2017). In this task, the neuronal A_1_R-deficient mice (*n* = 6) showed reduced TMT-induced freezing compared to control mice (A_1_R*^flx/flx^ n* = 11, Figure 3D). Freezing behavior enhanced during the trial because TMT concentration at the bottom of the cage increased with time. While this pattern was observed in both genotypes, the time-dependent increase in freezing was less pronounced in the A_1_R*^flx/flx^*;SNAP25*^Cre^* animals compared to control littermates (effect of the genotype × time interaction, *F* = 5.23; *P* = 0.032). Thus, neuronal A_1_R deficiency reduces olfaction-controlled behaviors in two paradigms based on unrelated behavioral functions. To exclude that neuronal A_1_R deficiency might have indirectly influenced olfaction tasks by altering behavioral functions, we tested A_1_R*^flx/flx^*;SNAP25*^Cre^* mice and A_1_R*^flx/flx^* littermates in an open field and elevated plus-maze test. However, no differences were detected between genotypes in either test (Supplementary Figures 3A–I), indicating that neuronal A_1_R alters olfaction but not novelty-induced anxiety and exploration.

### Neuroinflammation Leads to Mitral Cell Hyperexcitability That is Not Modulated by Neuronal Adenosine 1 Receptor

**FIGURE 4:**
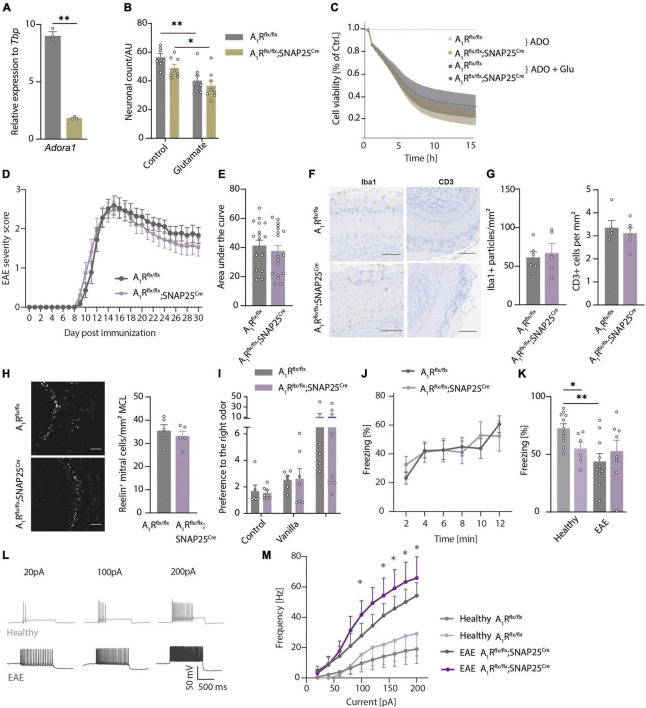
Neuroinflammation results in olfactory dysfunction and hyperexcitability that is not influenced by neuronal A_1_R. **(A)** *Adora1* expression in primary neuronal cultures of olfactory bulb neurons DIV 14–16 of A_1_R*^flx/flx^* (gray) and A_1_R*^flx/flx^*;SNAP25*^Cre^* (beige) (*n* = 3 per group). **(B)** Neuronal survival *in vitro* measured by MAP2-positive cells after treatment with 5 μM of glutamate for 6 h in A_1_R*^flx/flx^* and A_1_R*^flx/flx^*;SNAP25*^Cre^* (*n* = 3 biological replicates with 2–3 technical replicates). **(C)** Luminescence-based measurement of cell viability over 15 h treated with 10 μM of adenosine (ADO) and 5 μM of glutamate or medium as control (*n* = 4 biological replicates per group with at least 5 technical replicates each). **(D)** Clinical time course of experimental autoimmune encephalomyelitis (EAE) over 30 days (*n* = 18 per group) in A_1_R-proficient (A_1_R*^flx/flx^*) and A_1_R-deficient (A_1_R*^flx/flx^*;SNAP25*^Cre^*) mice. **(E)** Disease severity in EAE analyzed by area under the curve (BF_10_ = 0.384). **(F)** Representative images and **(G)** quantification of microglia activation measured by Iba1 (BF_10_ = 0.506), and T-cell infiltration evaluated by CD3-positive cells/mm^2^ (BF_10_ = 0.476) in the main olfactory bulb in coronal slices (*n* = 5 per group). **(H)** Reelin-positive mitral cells per mm mitral cell layer at day 30 post-immunization (p.i.) (*n* = 5 per group, BF_10_ = 0.673). **(I)** Olfactory detection test at the early phase of EAE (day 10–12 p.i.) in A_1_R*^flx/flx^ n* = 6 and A_1_R*^flx/flx^*SNAP25*^Cre^ n* = 7. As mice were exposed to odors at two sides of the cage (first trial empty control, second trial vanilla on the right side, third trial almond on the left side where vanilla was reintroduced on the right side), preference to one side of the cage has been measured by means of the ratio between the sides. **(J)** TMT-based olfactory testing measured by the percentage of freezing in bins of 2 min in EAE (A_1_R*^flx/flx^ n* = 10 and A_1_R*^flx/flx^*;SNAP25*^Cre^ n* = 8). **(K)** TMT-based olfactory behavioral test in healthy animals and at the early phase of EAE. Percentage of freezing after 10 min in A_1_R*^flx/flx^* (healthy *n* = 11 in light gray, EAE *n* = 10 in gray) and A_1_R*^flx/flx^*;SNAP25*^Cre^* mice (healthy *n* = 6 in light lavender, EAE *n* = 8 in lavender). **(L)** Representative traces of mitral cell action potentials in healthy control (light gray) and acute phase of EAE (dark gray). **(M)** Increase of frequency of action potentials of mitral cells depending on the injected current in healthy (*n* = 7) and acute EAE (*n* = 5) slice preparations of A_1_R*^flx/flx^* (healthy *n* = 7; EAE *n* = 5) and A_1_R*^flx/flx^*;SNAP25*^Cre^* (healthy *n* = 5; EAE *n* = 7) mice, analyzed by Mann–Whitney *U*-test. Statistical analysis was performed by two-way ANOVA in repetitive measurements **(C,D,J,L,M)** and unpaired *t*-test, if not stated otherwise; *P < 0.05, ^**^P < 0.01.

We demonstrated that mitral cells of the olfactory bulb express A_1_R and their activation leads to hyperpolarization (Rotermund et al., 2018) and reduction of calcium influx and glutamate release (Schulz et al., 2018). To dissect whether A_1_R-dependent effects could potentially counteract excessive glutamate release, hyperexcitability, and neuronal injury during neuroinflammation, we utilized primary cultures of olfactory bulb neuronal cells of A_1_R-proficient and A_1_R-deficient embryos. We detected *Adora1* expression by qRT-PCR in olfactory bulb primary neurons during neuronal differentiation at days *in vitro* (DIV) 7, 14, and 21 showing the peak of expression after 14 days (*n* = 3 per group, Supplementary Figure 4A). Furthermore, we confirmed the lack of *Adora1* transcripts in neuronal cultures dissected from A_1_R*^flx/flx^*;SNAP25*^Cre^* compared to wild types (A_1_R*^flx/flx^*) (*n* = 3 per group, Figure 4A). We confirmed the results by demonstrating a significant reduction of immunofluorescence A_1_R staining in A_1_R*^flx/flx^*;SNAP25*^Cre^* in comparison to wildtype cultures (*n* = 4 biological replicates per group with two technical replicates each, Supplementary Figure 4B). Upon the application of glutamic acid, neuronal survival was reduced to a similar extent in wildtype and A_1_R*^flx/flx^*;SNAP25*^Cre^* cultures as measured by counting MAP2-positive neurons (*n* = 3 biological replicates with 2–3 technical replicates, Figure 4B). By measuring the cell viability longitudinally over 15 h using a bioluminescence method (Realtime glo, Promega^®^), glutamic acid treatment led to a decrease irrespective of the genotype (*n* = 4 biological replicates per group with at least 5 technical replicates each, Figure 4C). Similar results were obtained by applying the A_1_R agonist 2-chloro-N6-cyclopentyladenosine (CCPA) to wildtype neuronal cultures (*n* = 4 biological replicates with at least 5 technical replicates each, Supplementary Figure 4C).

Next, we asked whether the A_1_R network of the olfactory bulb modulates neuronal survival during neuroinflammation in the EAE mouse model. However, A_1_R*^flx/flx^*;SNAP25*^Cre^* mice did not show a different disease course (*n* = 18 per group, Figure 4D), area under the curve (Figure 4E), onset (Supplementary Figure 4D), or final score at day 30 post-immunization (p.i.) (Supplementary Figure 4E) in comparison to A_1_R*^flx/flx^* littermate control mice. Also, on a neuropathological level, neither the microglial activation nor the amount of immune cell infiltration of T cells differed in the olfactory bulbs of the two genotypes in the chronic phase of EAE (*n* = 5 per group, Figures 4F,G). We could also not detect the differences between the genotypes in the counts of Reelin-positive neurons in the mitral cell layer (*n* = 5 per group, Figure 4H and Supplementary Figure 4F) or NeuN-positive cells (mostly adult neurons; Supplementary Figure 4G) in all the recorded layers at day 15 p.i. or at day 30 p.i. of EAE.

Since the clinical EAE score mainly reflects motor disabilities due to inflammation-induced injury of the spinal cord, we next tested whether inflammation functionally impairs olfaction. Thus, we assessed the animals in olfactory behavior using the odor detection test (A_1_R*^flx/flx^ n* = 6 and A_1_R*^flx/flx^*SNAP25*^Cre^ n* = 7, Figure 4I) and the TMT-based olfactory test (A_1_R*^flx/flx^ n* = 10 and A_1_R*^flx/flx^*;SNAP25*^Cre^ n* = 8, Figure 4J). We tested the animals at the early phase (EAE day 10–12 p.i.) of the disease to avoid paralysis-associated immobility which could compromise testing. Of note, in contrast to the reduced sensory performance of healthy neuron-specific A_1_R-deficient animals in olfactory tests, we detected no differences between A_1_R-deficient and wildtype EAE mice in their ability to detect the odor of vanilla and almond in the olfactory detection test (Figure 4I) and in their freezing response in TMT-based olfactory testing during EAE (Figure 4J). Neuroinflammation impaired olfactory function in the A_1_R-proficient mice in comparison to healthy condition (healthy *n* = 11, EAE *n* = 10) but was not further modulated in the A_1_R-deficient mice A_1_R*^flx/flx^*;SNAP25*^Cre^* (healthy *n* = 6, EAE *n* = 8, Figure 4K).

As extracellular concentrations of ATP and adenosine are elevated during inflammatory conditions (Cauwels et al., 2014; Di Virgilio et al., 2020), we explored a possible functional impact of A_1_R on excitability during inflammatory conditions, the acute phase of the EAE model. The analysis of mitral cell membrane parameters by patch-clamp recordings revealed an increase in the slope of the F/I plot in the brain slices of diseased animals (EAE day 13–16 p.i.) indicating higher excitability of the neurons in the inflammatory environment (Figures 4L,M). This increase, however, did not differ between A_1_R*^flx/flx^*;SNAP25*^Cre^* and A_1_R*^flx/flx^* controls, arguing against a pivotal role of neuronal A_1_R in controlling mitral cell excitability during inflammation (Figure 4M). In addition, we could not reveal an altered resting membrane potential (Supplementary Figure 4H), rheobase (Supplementary Figure 4I), or modulation of adenosine-induced outward current (Supplementary Figure 4J) due to neuroinflammation in both genotypes (measured at the acute phase of EAE day 13–16 p.i.). Taken together, we could detect inflammation-induced olfactory impairment and hyperexcitability that is, however, not functionally counteracted by A_1_R.

## Discussion

Adenosine is an important neuromodulator of intercellular communication in the olfactory bulb and is also involved in inflammatory conditions (Fredholm, 2014; Liu and Xia, 2015; Santiago et al., 2020). Here, we investigated to what extent the olfactory bulb is sensitive to inflammation-induced neurodegeneration and whether neuronal function, cellular integrity, and olfactory behavior can be modulated by the activity of A_1_R.

In our previous study (Schulz et al., 2018), we already demonstrated that constitutive A_1_R-deficient animals perform differently than wildtype animals in an olfactory-guided behavior test. Since adenosine is a powerful modulator of metabolism, such as thermoregulation and obesity (Carlin et al., 2018; Futatsuki et al., 2018; Gnad et al., 2020), we could not exclude that the observed difference in the buried food test was due to an altered metabolic state with different motivation of the animals to gather food. In contrast, the olfactory tests in the present study with neuron-specific A_1_R-deficient mice enabled us to show the importance of neuronal A_1_R for the olfactory sensory pathway with non-food-related olfactory tests. As the Snap25 promotor was used to drive Cre expression in neurons that is widely expressed throughout the brain, we cannot completely exclude the impact of other brain regions interconnected with the olfactory sensory pathway on olfactory behavior in this study. However, we used two different olfactory tests to confirm the specificity of the A_1_R effect on olfaction. The odor TMT has been used in several studies and the circuits activated by this odor are well understood (Kobayakawa et al., 2007; Saito et al., 2017; Sakano, 2020). We also excluded confounding changes in anxiety and curiosity in the neuron-specific A_1_R-deficient mice that confirmed previous results using global A_1_R-deficient mice (Chasse et al., 2021), whereas other studies have reported increased anxiety in A_1_R-deficient mice (Johansson et al., 2001; Giménez-Llort et al., 2002).

On the cellular level, immunostainings showed a reduced number of glutamatergic synapses in the external plexiform layer, and recordings of spontaneous PSCs in granule cells revealed a lower input frequency in the A_1_R*^flx/flx^*;SNAP25*^Cre^* mice. The main driver of granule cell activity is glutamate release from mitral and tufted cells (Egger and Urban, 2006; Schoppa, 2006), albeit granule cells also receive GABAergic inputs from different intra-bulbar sources, such as deep short axon cells and blanes cells or from the cortical feedback projections (Pressler and Strowbridge, 2006; Boyd et al., 2012; Nunez-Parra et al., 2013). However, since most of the inputs recorded in mature granule cells are excitatory (Carleton et al., 2003), we presume that the reduction of the number of postsynaptic currents in A_1_R-deficient granule cells is likely caused by a lack of glutamatergic synapses. However, we cannot entirely rule out a reduction of GABAergic inputs in knockout animals. Recent data describe A_2A_R-dependent development of GABAergic synapses in the hippocampal neurons (Gomez-Castro et al., 2021), implying a role of adenosine signaling not only in homeostatic plasticity but also during neurodevelopment. Whether the observed alteration in synaptic connections in the external layer of the olfactory bulb is due to an imbalance of adenosine signaling during the development has to be determined. However, we could not find pronounced differences in the membrane properties of mitral cells in neuron-specific A_1_R-deficient mice in comparison to wildtype controls. The application of adenosine was able to reduce spontaneous synaptic transmission in wildtype animals but not in A_1_R-deficient mice, as shown by the recordings of postsynaptic currents in the mitral cells and granule cells. The majority of synaptic connections in the olfactory bulb circuitry are formed between the principal neurons and axonless GABAergic interneurons, such as periglomerular cells, granule cells, and parvalbumin cells and therefore are reciprocal dendro-dendritic synapses (Crespo et al., 2013; Burton, 2017; Li et al., 2020). Consistent with our previous studies showing that neuronal A1R expression is restricted to mitral and tufted cells in the olfactory bulb and that the recurrent DDI of mitral cells by granule cells and parvalbumin interneurons is reduced by A_1_R signaling (Schulz et al., 2018), we conclude that adenosine modulates recurrent MC-GC synapses. However, an impact on other cell types and synapses in the circuitry cannot be ruled out. As inhibitory circuits play an important role in shaping odor representation in the olfactory bulb (Burton, 2017), alterations at this level of processing could impact olfactory function. Indeed, direct evidence for the impact of DDI on olfactory behavior exists for the granule cell–mitral cell interaction (Abraham et al., 2010; Kato et al., 2013; Miyamichi et al., 2013; Nunes and Kuner, 2015, 2018). Neuronal A_1_R-deficient mice showed olfactory dysfunction as shown in our two-sided odor exposition test using the odors of vanilla and almond. Of note, mice showed avoidance behavior when exposed to almond independent of their genotype. The aversive effect could have been caused by high concentrations of the almond flavor (Yang and Crawley, 2009; Islam et al., 2018). As both, wildtype littermates and A_1_R*^flx/flx^*;SNAP25*^Cre^* mice avoided the almond flavor, this suggested that neuronal A1R deletion impairs but does not abolish olfaction.

Notably, neuroinflammation results in neuronal injury that not only manifests as motor but also as olfactory dysfunction. In the TMT-based olfactory test, the EAE model reproduces human MS in which olfactory dysfunctions have been recorded (Goektas et al., 2011; Schmidt et al., 2017). The olfactory detection test however, exhibited high variability in preference to the odor in the EAE group in comparison to the healthy group and to the TMT-based analysis. We also reveal a hyperexcitability of mitral cells in EAE animals at the cellular level. Inflammation not only induces excitotoxicity by increasing the glutamatergic neurotransmission and concomitantly inducing the calcium overload of neurons, but also affects the expression and function of different classes of ion channels regulating the cellular ionic homeostasis (Friese et al., 2014). RNA expression data in MS brains indicate the dysregulation of a multitude of ion channels in inflamed brain areas, among those two-pore potassium channel TREK2, voltage-activated sodium channels Nav1.2 (Schattling et al., 2016), potassium channels Kv11.3 (erg), and calcium-activated potassium channel K_*Ca*_2.3 (Boscia et al., 2021). This could thereby affect all facets of neuronal homeostasis from resting membrane potential generation to action potential initiation and propagation. Since we blocked glutamate receptors during the examination of mitral cell excitability, we assume that the increase in gain in cells from EAE animals is not due to an acute increase of glutamatergic transmission but rather results from chronic inflammation-induced dysregulation of mitral cell ion channels. However, apart from the hyperexcitability, we could not find other apparent alterations in physiological parameters, such as resting membrane potential or whole cell currents in our recordings.

As inflammation is linked to increased concentrations of extracellular ATP and adenosine (Di Virgilio et al., 2020), purinergic receptor-mediated signaling could be involved in the modulation of neurodegeneration. Thus, A_1_R-dependent signaling in neurons has the potential to counteract hyperexcitability and excitotoxicity, as its activity results in hyperpolarization and inhibition of calcium-entry *via* Ca_*v*_ (Schampel and Kuerten, 2017). However, our *in vitro* studies with olfactory bulb neurons from neuron-specific A_1_R-deficient animals and respective controls, as well as activation of the neuronal A_1_R failed to rescue neurons from glutamate excitotoxicity. In contrast, the overexpression of A_1_R in neurons by transfection had a positive effect on cell survival in an NMDA-based excitotoxicity assay (Serchov et al., 2012). Expression changes of glutamate receptors after the activation of A_1_R could also interact with these results (Chen et al., 2015). The authors of this study analyzing the NMDA-based excitotoxicity used an inducible expression system that might be able to reveal acute effects of A_1_R-dependent signaling, whereas our knockout model might be compromised by developmental effects. Similarly, the hyperexcitability of mitral cells was modified by a lack of A_1_R expression. Finally, in our *in vivo* studies, we could not reveal an A_1_R-dependent neuroprotective phenotype during neuroinflammation. Thus, the potential beneficial effect of A_1_R-signaling in neurons is likely overruled and fails to protect olfactory neurons from hyperexcitability, calcium overload, and injury during neuroinflammation.

A possible explanation for the lack of neuroprotective function *via* A_1_R activation could be the affinity of the P1 receptors to their ligand adenosine depending on the extracellular concentration. In contrast to the low-affinity receptors A_2B_R and A_3_R, the A_1_R and the A_2A_R show a high affinity to adenosine. While activation of A_1_R is already present at low extracellular concentrations of adenosine (0.3–3 nM), which are mainly found under physiological conditions, activation of the A_2A_R requires a 10-fold rise of the extracellular adenosine concentration (Stockwell et al., 2017). According to these pharmacological properties, the inhibitory and neuroprotective capacity of A_1_R could be overruled by the action of A_2A_R that acts as a counterpart. This could result in a dominance of A_2A_R-mediated signaling in EAE. Another explanation could be the internalization of A_1_R due to chronic activation (Klaasse et al., 2008). Several studies show that during chronic caffeine consumption, tolerance of A_1_R to caffeine occurs by internalization. In parallel, the A_2A_R becomes predominant in the action of caffeine (Nehlig et al., 1992). In conclusion, the high level of extracellular adenosine during neuroinflammation could paradoxically lead to the suppression of neuroprotective adenosine-dependent downstream effects in neurons.

Moreover, the observations by Tsutsui et al. (2004) that the constitutive global A_1_R knockout exacerbates EAE severity is in agreement with our results, as they attribute this effect to the immunosuppressive function of A_1_R-signaling (Tsutsui et al., 2004). As A_1_R is mainly expressed on immature dendritic cells, macrophages, and neutrophils in which its activity suppresses their activation, an A_1_R deficiency would be expected to exacerbate CNS inflammatory disease (Cekic and Linden, 2016). The results obtained with the neuron-specific A1R knockout have some limitations. Combining the low throughput patch clamp-technique with the animal model of EAE revealed first insights into the olfactory bulb-specific response to inflammation. However, in some analyses Bayesian statistics, applied to evaluate negative results, revealed Bayes factors greater than 0.33. This indicates that we cannot entirely exclude that the absence of an effect is due to low statistical power. At this point, however, data of the global A_1_R knockout and our data with the neuron-specific A_1_R knockout suggest that neuronally expressed A_1_R plays a minor role in resilience to inflammation in the EAE model.

## Materials and Methods

### Mice

All mice [*C57Bl/6J* wild type (The Jackson Laboratory), *A_1_R*^flx/flx^** mice (provided by Frank Kirchhoff, Homburg, Germany) (Scammell et al., 2003), *SNAP25-IRES-Cre* mice (The Jackson Laboratory) (Harris et al., 2014), “*Tagger*” knockin mouse line provided by Walker Scot Jackson (Kaczmarczyk et al., 2019)] were kept under specific pathogen-free conditions in the central animal facility of the University Medical Center Hamburg-Eppendorf, Hamburg, Germany. Neuron-specific knockout mice with or without fluorescent nuclear tag (*A_1_R *^flx/flx^*;SNAP25*^Cre^**, *A_1_R *^flx/flx^*;Tag;SNAP25*^Cre^**) were generated by crossing *A_1_R*^flx/flx^** with *SNAP25-IRES-Cre* with or without the “*Tagger*” reporter mouse line (Kaczmarczyk et al., 2019). Mice were housed in a facility at 24 ± 2°C and 55–65% humidity with a 12-h light/dark cycle and had free access to food and water. Sex and age-matched adult animals (8–16 weeks of age) were used in all experiments. For behavioral experiments, animals were housed in an inverted light/dark cycle to enable the performance of the animal experiments in the animal’s active day phase. Animals were adapted to the inverted light/dark cycle at least 3 weeks in advance. Experiments were carried out under red light and light exposure of less than 25 lux.

### Mouse Tissue Preparation for Acute Slices

Olfactory bulbs were dissected, and horizontal brain slices were prepared as described before (Fischer et al., 2020). Briefly, olfactory bulbs were quickly transferred into a chilled slicing artificial cerebrospinal fluid (ACSF) that contained (in mM): NaCl, 83; NaHCO_3_, 26.2; NaH_2_PO_4_, 1; KCl, 2.5; sucrose, 70; D-glucose, 20; CaCl_2_, 0.5; MgSO_4_, 2.5. About 200 μm thick acute slices of the bulbs were cut using a vibrating blade microtome (Leica VT1200S, Bensheim, Germany). The brain slices were stored in ACSF containing (in mM): NaCl, 120; NaHCO_3_, 26; NaH_2_PO_4_, 1; KCl, 2.5; D-glucose, 2.8; CaCl_2_, 2; MgCl_2_, 1. Storage lasted for 30 min at 30°C and at least 30 min at RT before starting the experiments. The ACSF was continuously gassed with carbogen (95% of O_2_ and 5% of CO_2_; buffered to pH 7.4 with CO_2_/bicarbonate).

### Electrophysiological Recordings

Mitral cells of the main olfactory bulb were investigated using the patch clamp technique (EPC 10 amplifier, HEKA Elektronik GmbH Reutlingen, Germany). Throughout the experiments, the brain slices were superfused with ACSF. Drugs were applied with the perfusion system driven by a peristaltic pump (Reglo, Ismatec, Wertheim, Germany) at a flow rate of 2 ml min^–1^. Application bars reflect the time window during which drug-containing ACSF is present in the bath. The whole cell configuration was employed using patch pipettes with a resistance of ∼3–5 MΩ for mitral cells and ∼4–6 MΩ for granule cells. Recordings were digitized at 10–20 kHz and filtered (Bessel filter, 22 kHz). The standard pipette solution contained (in mM) the following: NaCl, 10; potassium gluconate, 105; K_3–_-citrate, 20; Hepes, 10; MgCl_2_, 0.5; Mg-ATP, 3; Na-GTP, 0.5; EGTA, 0.25. Cells were held at −50 mV in voltage clamp recordings and from −50 to −55 mV in current clamp recordings by appropriate current injection. High chloride pipette solution contained (in mM) the following: CsCl, 120; Hepes, 10; EGTA, 0.2; MgCl_2_, 2; CaCl_2_, 0.075; Na-ATP, 2; Na-GTP, 0.5; 4-AP, 5; TEA-Cl, 20. Cells were held at −70 mV in a voltage clamp recording with high chloride pipette solution. For mitral cell membrane property measurements, only cells of confirmed wildtype and knockout phenotype, that is, only cells that showed adenosine-induced outward current in wildtype and absence of adenosine-induced outward current in knockout animals, respectively, were included in the analysis. Series resistance (R_*series*_) was monitored during the recording, and cells exceeding 20 MΩ and/or a change of 20% in R_*series*_ were excluded from the analysis. Cells exceeding an input resistance of 500 MΩ were also excluded from the analysis. Current clamp recordings for F/I plot were performed in the presence of 10 μM of NBQX, 50 μM of DAP-V, and 5 μM of gabazine. For the recording of current-voltage relationship in the voltage clamp, 0.5 μM of TTX was added, currents displayed are not leak subtracted, and voltages displayed are not corrected for liquid junction potential (−17 mV for potassium glutamate intracellular solution). For recordings of the adenosine effect in granule cells, series resistance was monitored and cells with R_*series*_ higher than 30 MΩ and/or a change of 20% during the recording were excluded from the analysis. Electrophysiological data were analyzed using *Mini Analysis* (Synaptosoft, Fort Lee, NJ, United States), *ClampFit* (Molecular Devices), *Review* (Bruxton), and *OriginPro* (Northampton, MA, United States).

### Behavioral Tests

Mice were transferred from the breeding facility into a vivarium and maintained under standard housing with an inverted 12h/12h-light/dark cycle (light off at 8:00 am), i.e., experiments were transduced during the dark phase. All tests were performed with 10–16-week-old mice in a room next to the vivarium illuminated with dim red light. Tests started and ended at least 2 h after light offset and 3 h before light onset. The experimental material was cleaned with soap, water, and ethanol (70%) before and after each contact with an animal. All tests were video-recorded. Tracks representing the position of the mice were created and analyzed with the software, *EthoVision* (Noldus) as described (Freitag et al., 2003). Analysis of behavior with the software, *The Observer* (Noldus) was performed by a trained experimenter blind to the genotypes. The experimenter trained himself until he repeatedly scored at least 90% of consistency between two analyses performed at different times on the same mouse, as calculated with the Reliability Test provided by *The Observer* (having 1 s as maximal time discrepancy between two evaluations).

### Olfactory Detection Test

Mice were examined in their ability to detect two new odors (vanilla and almond, Dr. Oetker) sequentially during a two-sided odor exposition in a test cage (Type II long, 325 mm × 170 mm × 140 mm) under red light. Mice were exposed in three trials of 4 min to either the empty tube (control) or a tube filled with a Whatman paper (0.6 cm Ø) containing 5 μl of the test odor of vanilla or almond. The tubes were placed at two openings in the middle of the short sides of the cage at a height of 3 cm from the bottom. The control tubes and test tubes were exchanged simultaneously on both sides of the cages to minimize a handling bias. Time sniffing at the tube was automatically measured by the video tracking system, *EthoVision* and offline manually by a trained experimenter blinded to experimental conditions using the software *The Observer*. Mice were habituated to the environment 5 days before the testing. This experiment was performed in healthy conditions with two cohorts with similar results. The EAE mice were tested at an early phase of EAE (severity score ≤1.5).

### Trimethylthiazoline-Induced Freezing

A Whatman paper of 1 cm × 2 cm nourished with 10 μl of 15% of TMT was fixed to the side of a testcage (325 mm × 170 mm × 140 mm). Mice were placed in the test cage immediately. The cages were closed to avoid diffusion of the odor and exposition to other environmental stimuli. Reaction to the rising odor concentration was measured by the time of freezing in a trial of 12 min by a video tracking system (*EthoVision*) and manually by a rater, blinded to the genotype of the tested animal. Analyses were done under red light <20 lux. Mice were tested in a healthy condition or at an early phase of EAE (severity score ≤1.5).

### Open Field

The open-field test was performed in a box (50 cm × 50 cm and 40 cm high) illuminated with white light (10 lux). Mice were placed in one corner of the box and their behavior was analyzed for 15 min. Distance moved, mean minimal distance to the wall, and time spent in the center (an imaginary 25 cm × 25 cm square in the middle of the arena) were analyzed with the *EthoVision* software. In contrast, the parameters rearing on the wall (mouse stands on hind limbs and touches the wall with at least one forepaw) and self-grooming were analyzed with *The Observer*.

### Elevated Plus-Maze

The maze has the shape of a plus with four 30 cm long and 5 cm wide arms, connected by a squared center (5 cm × 5 cm). Two opposing arms are bordered by 15 cm high walls (closed arms), whereas 2 mm rim borders the other two arms (open arms). The maze was elevated 75 cm from the floor, and an infrared camera was allowed for video recording. The mouse was placed in the center facing one open arm and left in the maze for 5 min. The following parameters were analyzed with *The Observer*: entries into open and closed arms (calculated when all four paws were on an arm), total transitions (sum of entries into open and closed arms), entries into edges of open arms (calculated when the mouse reaches with its snout the edge of an open arm), latency to enter into open arms, latency to reach the edge of an open arm, stretched attend posture toward the open arm, rearing, self-grooming, head dips from “protected” area (head movements over the side of an open arm with the snout pointing downward while the mouse remains in the center or closed arm), and head dips from “unprotected” area (head dips are done as the mouse is on the open arms).

### Experimental Autoimmune Encephalomyelitis Induction and Scoring

For the induction of EAE, the mice were anesthetized with 1% of isoflurane to 2% v/v oxygen and immunized subcutaneously with 200 μg of myelin oligodendrocyte glycoprotein 35–55 (MOG_35_–_55_) peptide (peptides elephants) in emulsion with complete Freund’s adjuvant (BD) containing 4 mg ml^–1^ of *Mycobacterium tuberculosis* (BD). This is followed by the administration of 200 ng of PTX in PBS on the day of immunization and at day 2 after immunization. Animals were assessed daily. Clinical signs of EAE were scored as follows: 0, no clinical deficits; 1, tail weakness; 2, hind limb paresis; 3, partial hind limb paralysis; 3.5, full hind limb paralysis; 4, full hind limb paralysis, and forelimb paresis; 5, premorbid, or dead. Animals reaching a clinical score ≥≥4 had to be killed according to the regulations of the Animal Welfare Act.

### Mouse Tissue Preparation and Immunohistochemistry

Mice were anesthetized intraperitoneally with 100 μl of solution [10 mg ml^–1^ of esketamine hydrochloride (Pfizer), 1.6 mg ml^–1^ of xylazine hydrochloride (Bayer) dissolved in water] per 10 g of body weight. For histopathology and immunohistochemistry, the mice were perfused with 4% of paraformaldehyde (PFA), the brain tissue was dissected, fixed for 4 h with 4% of PFA, and then transferred to 30% of sucrose in PBS at 4°C. Main olfactory bulbs were sliced in 12 μm sections in coronal orientation with a freezing microtome (Leica Jung CM3000) and stored in a cryoprotective medium (Tissue Tek^®^ from Sakura) at −80°C. Cryosections of olfactory bulb were incubated in 10% of normal donkey serum containing 0.1% of Triton X-100 and were subsequently stained with antibodies against the following: CNPase (mouse, Sigma-Aldrich; Cat# C5922, RRID: AB_476854), GFAP (chicken, Millipore; Cat# AB5541, RRID: AB_177521), HA-tag (rat, Sigma Aldrich; Cat# 11867423001, RRID: AB_390918), HuC/D (mouse, Molecular Probes; Cat# A-21271, RRID: AB_221448), Iba1 (rabbit, Wako Shibayagi; Cat# 019-19741, RRID: AB_839504), NeuN (chicken, Millipore; Cat# ABN91, RRID: AB_11205760), Reelin (mouse, Millipore; Cat# MAB5364, RRID: AB_2179313), and TH (mouse, Synaptic Systems; Cat# 213 104, RRID:AB_2619897). As secondary antibodies, we used Alexa Fluor 488–coupled donkey antibodies recognizing chicken IgG (1:600, Jackson Immunoresearch; Cat# 703-545-155, RRID: AB_2340375), rabbit IgG (1:600, Jackson Immunoresearch; Cat# 711-546-152, RRID: AB_2340619) and mouse IgG (1:600, Abcam; Cat# ab150105, RRID: AB_2732856), Alexa Fluor 647–coupled donkey antibodies recognizing rat IgG (1:600, Abcam; Abcam; Cat# ab150159, RRID: AB_2566823) and rabbit IgG (1:600, Abcam; Cat# ab150075, RRID: AB_2752244). To avoid background labeling when using anti-mouse primary antibodies in inflamed mouse tissue, we used a Fab fragment anti-mouse IgG (1:200, Jackson Immunoresearch, Cat# 715-007-003, RRID: AB_2307338) in 0.1% of Triton X-100 for 1 h before using the anti-mouse antibody. Images were acquired using a Zeiss LSM 700 confocal microscope. Samples were analyzed by the open-source package *Fiji* based on *ImageJ*. Reelin positive HuC/D positive and TH positive cells were counted manually restricted to the layer. The quantification of NeuN positive cells was automatized after binarization of the selected area using the threshold *triangle*.

### Histopathology

For histopathology of EAE mice, the brain tissue was fixed with 4% PFA as mentioned above. After 4 h of fixation, the tissue was stored in PBS, cast in paraffin, and stained according to the standard procedures of the UKE Mouse Pathology Facility. For orientation in the tissue, hematoxylin staining (blue color) was done, followed by anti-CD3 primary antibodies (rabbit IgG, Abcam Cat# ab16669, RRID: AB_443425) and anti-Iba1 (rabbit IgG, Fujifilm Wako Shibayagi; Cat# 019-19741, RRID: AB_839504) that were visualized using the avidin–biotin complex technique with 3,3′-diaminobenzidine (brown stain). Slides were imaged with a *NanoZoomer 2.0-RS* digital slide scanner. CD3 positive and Iba1 positive cells were analyzed using the *NDP.view2* software (Hamamatsu) and the open-source software *Qpath*.^1^

### Immunofluorescence

Olfactory bulb neurons (DIV 16–18) were fixed with 4% of PFA for 10 min at room temperature, permeabilized with 0.05% of Triton and blocked with 10% of normal donkey serum in PBS. Cells were incubated for 2 h with antibodies directed against A_1_R (rabbit, Novus Biologicals; Cat# NB300-549, RRID: AB_10002337) and microtubule-associated protein 2 (MAP2, chicken, 1:2500; Abcam; Cat# ab5392, RRID: AB_2138153). As secondary antibodies, we used Alexa Fluor 488–coupled donkey antibodies recognizing chicken IgG (1:600, Jackson; Cat# 703-545-155, RRID: AB_2340375) and Alexa Fluor 647–coupled donkey antibodies recognizing rabbit IgG (1:600, Abcam; Cat# ab150075, RRID: AB_2752244). Images were taken with a Zeiss LSM 700 confocal microscope.

### Primary Neuron Culture

Primary olfactory bulb cultures were prepared from E16.5 embryos. Olfactory bulb pieces were incubated in 0.05% of Trypsin-EDTA (Gibco) for 6 min at 37°C. Trypsination was stopped by DMEM-F12 containing 10% of FCS. Afterward, tissue was dissociated in HBSS and centrifuged for 2 min at 500 × *g*. The pellet was resuspended in primary growth medium (PGM), and cells were plated at 1 × 10^5^ per cm^2^ on poly-d-lysine-coated cell culture plates. We maintained cells in Primary Neuron Growth Medium BulletKit (PNGM, Lonza) at 37°C, 5% of CO_2_, and relative humidity of 98%. To inhibit glial proliferation, we added cytarabine (Sigma, 1 μM = AraC) and maintained cultures for 14–18 days *in vitro*.

### Cell Viability Assay

Olfactory bulb primary neuron cultures were seeded in 96-well cell culture plates (Greiner, 655094) coated with poly-d-lysine as described above. Cell viability was measured by non-lytic NanoLuc Luciferase reaction assay (Promega, RealTime-Glo™ MT cell viability assay, G9711) in a plate reader (Tecan Spark Cyto) over 24 h. Treatment was added 5 h after the substrate and enzyme of RealtimeGlo™ to stabilize the luciferase reaction.

### Quantitative Real-Time PCR

Isolated RNA was reversed transcribed to cDNA with the RevertAid H Minus First Strand cDNA Synthesis Kit (Thermo Fisher Scientific) according to the manufacturer’s instructions. Gene expression was analyzed by real-time PCR performed in an ABI Prism 7900 HT Fast Real-Time PCR System (Applied Biosystems) using TaqMan Gene Expression Assays (Thermo Fisher Scientific) for *Adora1* (Mm01308023_m1), *Adora2* (Mm00802075_m1) and *Tbp* (Mm00446971_m1), and *Cdhr1* (Mm00499982_m1) and *Tbp* (Mm01277042_m1). Gene expression was calculated as 2^–Δ*Ct*^ relative to *Tbp* as the endogenous control.

### Statistics

Experimental data were analyzed within the R environment using *Rstudio* (version 1.2.5033) and *GraphPad Prism* (version 9.3.1). The data are presented as mean ± SEM throughout, and differences between two experimental groups were determined using unpaired, two-tailed Student’s *t*-tests, and were FDR corrected for multiple comparisons or Mann–Whitney *U*-test according to normality distribution test by Shapiro–Wilk. Statistical analysis of the clinical scores in the EAE experiments was performed by applying the Mann–Whitney *U*-test to the AUCs for each animal. Repeated and paired measurements (trial and odor in the olfactory detection test, and time bins in the open field and TMT-induced freezing test) were analyzed by mixed two- or three-way ANOVA with genotype as a between-groups factor and the appropriate within-groups factor(s) followed by Dunnet’s *post-hoc* analysis when appropriate. Electrophysiological data are indicated as mean ± SEM and were analyzed by ORIGINPro software using Mann–Whitney and Wilcoxon ranking tests for unpaired samples and paired samples, respectively. Validity of negative results obtained in frequentist statistical approaches were tested by Bayesian statistics using JASP software (Keysers et al., 2020). Resulting Bayes factors (BF_10_) indicate the following on a linear scale: BF_10_ < 0.3 is the evidence of absence of an effect (samples are equal); 0.3 < BF_10_ < 3 is the absence of evidence (no final conclusion permissible); BF_10_ > 3 is the evidence of effect (samples are different). The exact number of experiments is provided in the figure legends. All tests were two-tailed, and significances are indicated by *P* < 0.05, *P* < 0.01, and *P* < 0.001.

## Data Availability Statement

The original contributions presented in this study are included in the article/Supplementary Material, further inquiries can be directed to the corresponding authors.

## Ethics Statement

All animal care and experimental procedures were performed according to institutional guidelines and conformed to requirements of the German Animal Welfare Act. All animal experiments were approved by the local ethics committee (Behörde für Soziales, Familie, Gesundheit und Verbraucherschutz in Hamburg: 122/17). We conducted all procedures in accordance with the ARRIVE guidelines (Kilkenny et al., 2010).

## Author Contributions

CS, DH, FM, and MF designed the experiments for the study and wrote the manuscript. CS, DH, KS, AO, A-LP, and FM analyzed the data. CS, KS, ST, DH, FM, A-LP, and SR performed experiments. All authors contributed to the article and approved the submitted version.

## Conflict of Interest

The authors declare that the research was conducted in the absence of any commercial or financial relationships that could be construed as a potential conflict of interest.

## Publisher’s Note

All claims expressed in this article are solely those of the authors and do not necessarily represent those of their affiliated organizations, or those of the publisher, the editors and the reviewers. Any product that may be evaluated in this article, or claim that may be made by its manufacturer, is not guaranteed or endorsed by the publisher.

## Footnotes

1
https://qupath.github.io/
