# Supplementary material for: Neuronal Adenosine A1 Receptor is Critical for Olfactory Function but Unable to Attenuate Olfactory Dysfunction in Neuroinflammation
Source: Front Cell Neurosci. 2022 Jun 30;16:912030. doi: 10.3389/fncel.2022.912030 (PMC9279574; doi:10.3389/fncel.2022.912030)
Supplement: Supplementary file 1 [file Data_Sheet_1.pdf]

Supplementary Figure 1

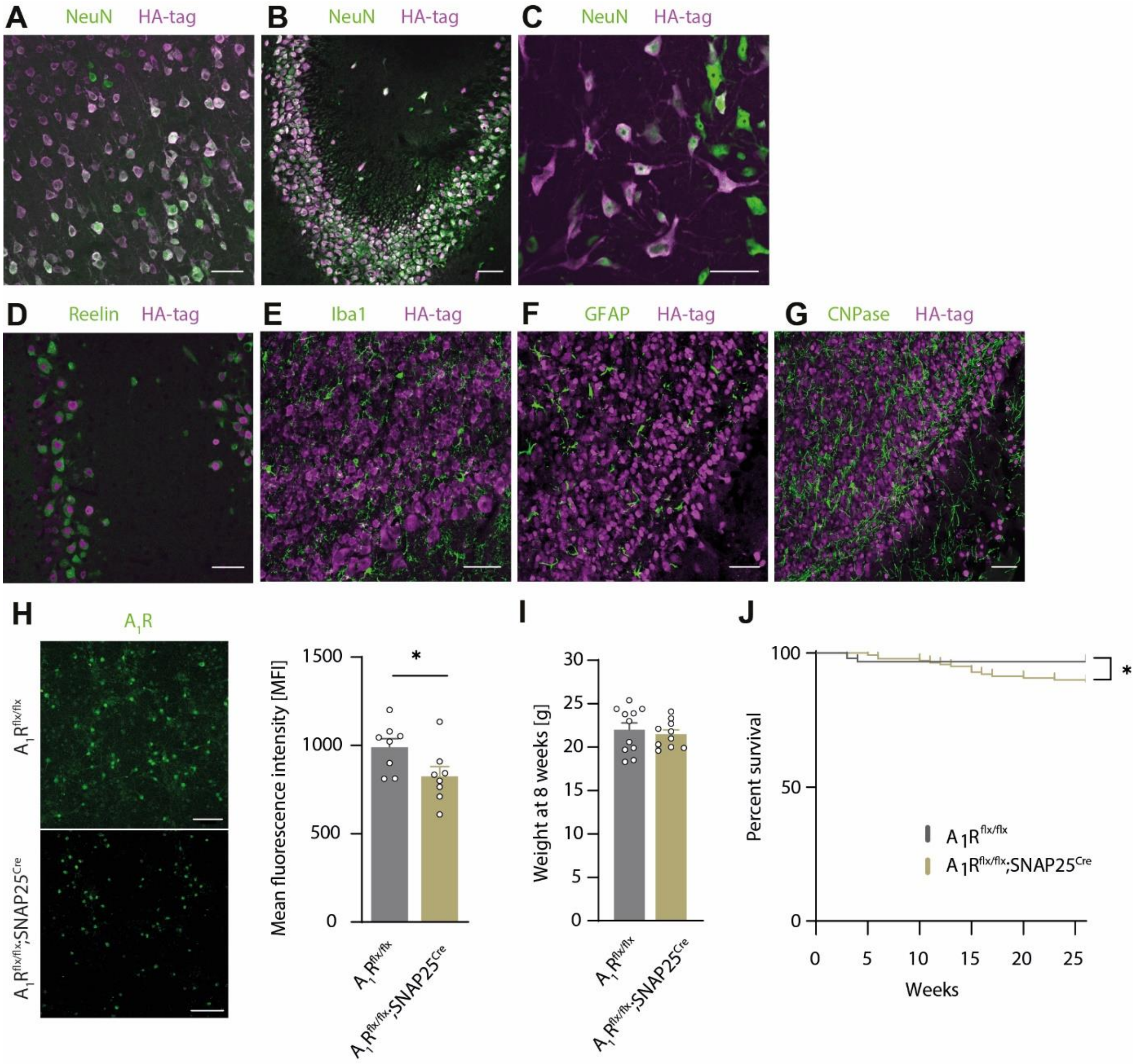

Supplementary Figure 1 | Characterization of $A_1R^{fl/fl}$;$SNAP25^{Cre}$ mice. (A–C) Reporter expression in neuron-specific mouse line $A_1R^{flx/flx}$;Tag;$SNAP25^{Cre}$ indicates a wide distribution of Cre expression in different brain regions (scale bars = 50 µm): **(A)** cortex, **(B)** hippocampus: CA3 region of the dentate gyrus and **(C)** spinal cord: motor neurons in the ventricular horn are shown. HA-tag counterstained with anti-HA antibody (magenta) labeling Cre expression cells, NeuN counterstaining (green) stains the majority of neurons in these areas. **(D)** Mitral cells and tufted cells were stained by marker Reelin (green) with Tag (magenta) in the main olfactory bulb in the $A_1R^{flx/flx}$;Tag;$SNAP25^{Cre}$. **(E–G)** Immunostaining of the HA-tag (magenta) in the $A_1R^{flx/flx}$;Tag;$SNAP25^{Cre}$ together with specific markers for **(E)** microglia (Iba1), **(F)** astrocytes (GFAP) and **(G)** oligodendrocytes (CNPase) in green (Scale bars = 50 µm). **(H)** Mean fluorescence intensity of the antibody directed against A_1R in $A_1R^{flx/flx}$ and $A_1R^{flx/flx}$;$SNAP25^{Cre}$ primary neuronal culture as representative images (left, scale bar = 100 µm) and quantification (right) (four biological replicates with two technical replicates each). **(I)** Mean weight of $A_1R^{flx/flx}$ and $A_1R^{flx/flx}$;$SNAP25^{Cre}$ mice at 8 weeks of age (BF_{10} = 0.327). **(J)** Kaplan-Meier survival curve of $A_1R^{flx/flx}$ (n = 139) and $A_1R^{flx/flx}$;$SNAP25^{Cre}$ (n = 170) animals until the age of 26 weeks. Log rank test were performed. *$P < 0.05$.
