# Supplementary material for: Neuronal Adenosine A1 Receptor is Critical for Olfactory Function but Unable to Attenuate Olfactory Dysfunction in Neuroinflammation
Source: Front Cell Neurosci. 2022 Jun 30;16:912030. doi: 10.3389/fncel.2022.912030 (PMC9279574; doi:10.3389/fncel.2022.912030)
Supplement: Supplementary file 2 [file Data_Sheet_2.pdf]

Supplementary Figure 2

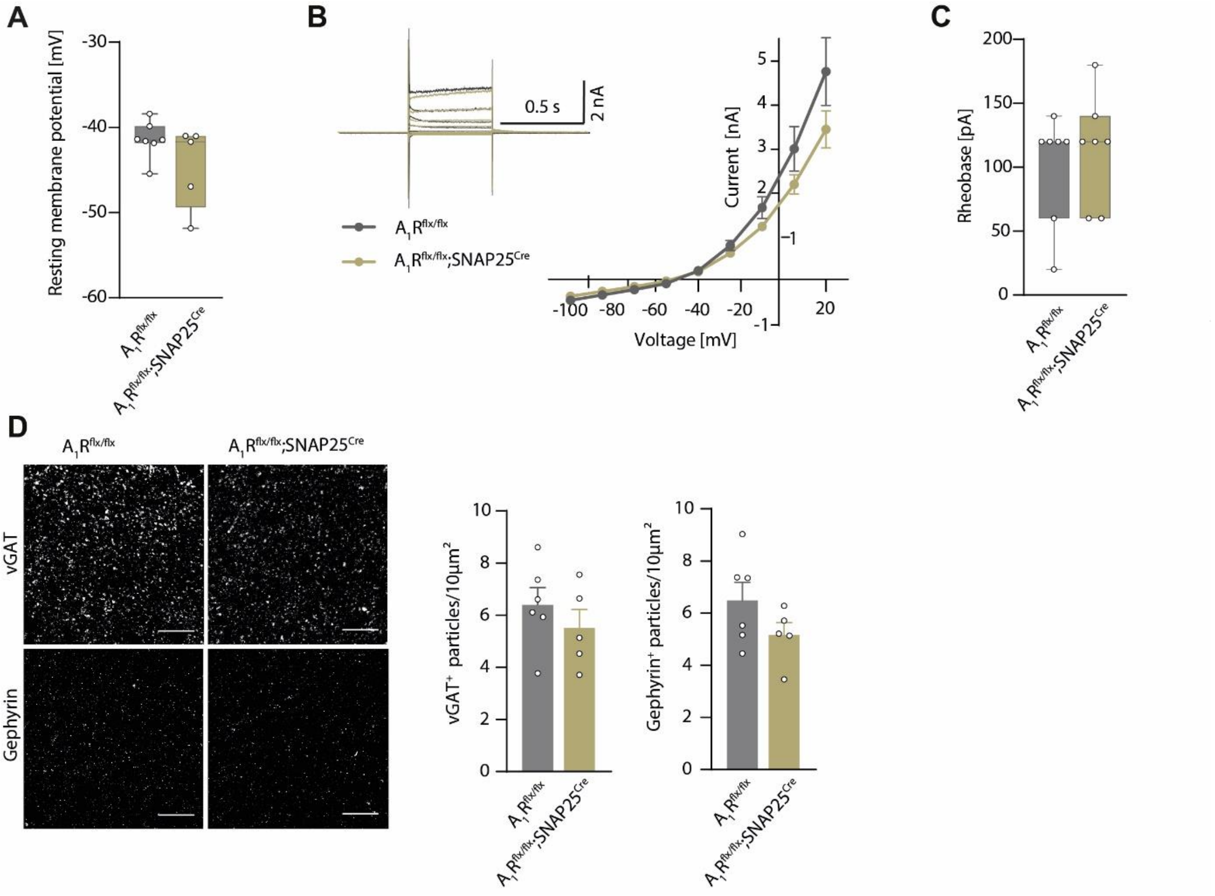

Supplementary Figure 2 | No apparent differences in membrane properties between A_1R-proficient and A_1R-deficient mitral cells. (A) Current-clamp recording of resting membrane potential of mitral cells in acute slices of A1R$^{flx/flx}$ and A1R$^{flx/flx}$;SNAP25^{Cre} animals. Measurement was performed immediately after establishing whole cell configuration, BF_{10} = 0.929. **(B)** Current-voltage relationship recorded in voltage clamp (from –100 to 20 mV in 15 mV steps, see inset) in whole cell conductance in mitral cells of both genotypes (BF_{10} = 0.514-0.998 for different voltage steps). **(C)** Firing behavior was analyzed by application of depolarizing current injections of consecutively higher amplitude. Rheobase shows the first current step resulting in a membrane depolarization beyond the action potential threshold in A_1R$^{flx/flx}$ (91 ± 17 pA, *n* = 7 slices of 5 mice) and A_1R$^{flx/flx}$;SNAP25^{Cre} (112 ± 23 pA, *n* = 5 slices of 4 mice) (BF_{10} = 0,559). In A–C only cells with phenotypically confirmed A_1R function or absence, respectively, as detected by application of adenosine during the later course of recording were considered. Displayed voltage is not corrected for liquid junction potential. Boxplots show mean to max values. **(D)** Representative images and quantification of inhibitory synaptic density in the external plexiform layer (EPL) of the main olfactory bulb in A_1R-proficient (A_1R$^{flx/flx}$) and A_1R-deficient (A_1R$^{flx/flx}$;SNAP25^{Cre}) mice. Immunohistochemical stainings comprised the presynaptic protein vGAT (BF_{10} = 0.622) and the postsynaptic protein gephyrin (BF_{10} = 0.917) in the EPL in A_1R$^{flx/flx}$ (*n* = 6) and A_1R$^{flx/flx}$;SNAP25^{Cre} (*n* = 5) (Scale bar = 20µm).
