# Supplementary material for: Neuronal Adenosine A1 Receptor is Critical for Olfactory Function but Unable to Attenuate Olfactory Dysfunction in Neuroinflammation
Source: Front Cell Neurosci. 2022 Jun 30;16:912030. doi: 10.3389/fncel.2022.912030 (PMC9279574; doi:10.3389/fncel.2022.912030)
Supplement: Supplementary file 3 [file Data_Sheet_3.pdf]

Supplementary Figure 3

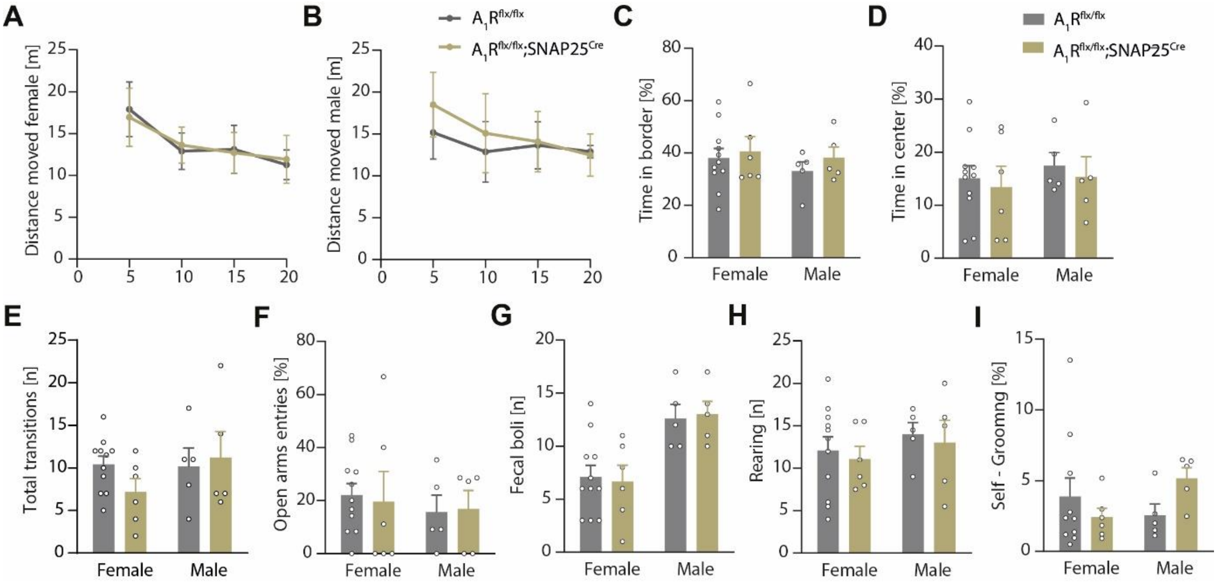

Supplementary Figure 3 | Neuronal A_1R does not affect exploratory and anxiety behaviors. Exploratory and anxiety behavior was analyzed in A_1R-proficient and A_1R-deficient mice. To reveal sex differences, animals were analyzed separately in $A_1R^{flx/flx}$ (*n(f)*= 11, *n(m)* = 5) and $A_1R^{flx/flx}$;SNAP25^{Cre} (*n(f)*= 6, *n(m)* = 5). **(A–D)** Open field test. The distance animals moved in the open field measured in meter in female **(A)** and male **(B)** mice of $A_1R^{flx/flx}$ and $A_1R^{flx/flx}$;SNAP25^{Cre}. Repeated measures were analyzed by two way ANOVA. **(C)** Percentage of time mice spent at the border in the open field test (BF_{10}*(f)* = 0.445, BF_{10}*(m)* = 0.647). **(D)** Percentage of time mice spent in the center of the open field (BF_{10}*(f)* = 0.452, BF_{10}*(m)* = 0.527). **(E-I)** Elevated maze test. **(E)** Number of transitions between experimental arms in the elevated plus maze (BF_{10}*(f)* = 1.299, BF_{10}*(m)* = 0.504). **(F)** Percentage of entries in the arms without walls in the elevated plus maze (BF_{10}*(f)* = 0.455, BF_{10}*(m)* = 0.495). **(G)** Number of fecal boli counted in the open field test indicating anxiety behavior (BF_{10}*(f)* = 0.440, BF_{10}*(m)* = 0.500). **(H)** Number of rearing events in male and female mice analyzed in the elevated plus maze (BF_{10}*(f)* = 0.445, BF_{10}*(m)* = 0.510). **(I)** Percentage of time per trial demonstrating behavior of self-grooming during the elevated plus maze testing (BF_{10}*(f)* = 0.545, BF_{10}*(m)* = 1.143).
