# Supplementary material for: Neuronal Adenosine A1 Receptor is Critical for Olfactory Function but Unable to Attenuate Olfactory Dysfunction in Neuroinflammation
Source: Front Cell Neurosci. 2022 Jun 30;16:912030. doi: 10.3389/fncel.2022.912030 (PMC9279574; doi:10.3389/fncel.2022.912030)
Supplement: Supplementary file 4 [file Data_Sheet_4.pdf]

Supplementary Figure 4

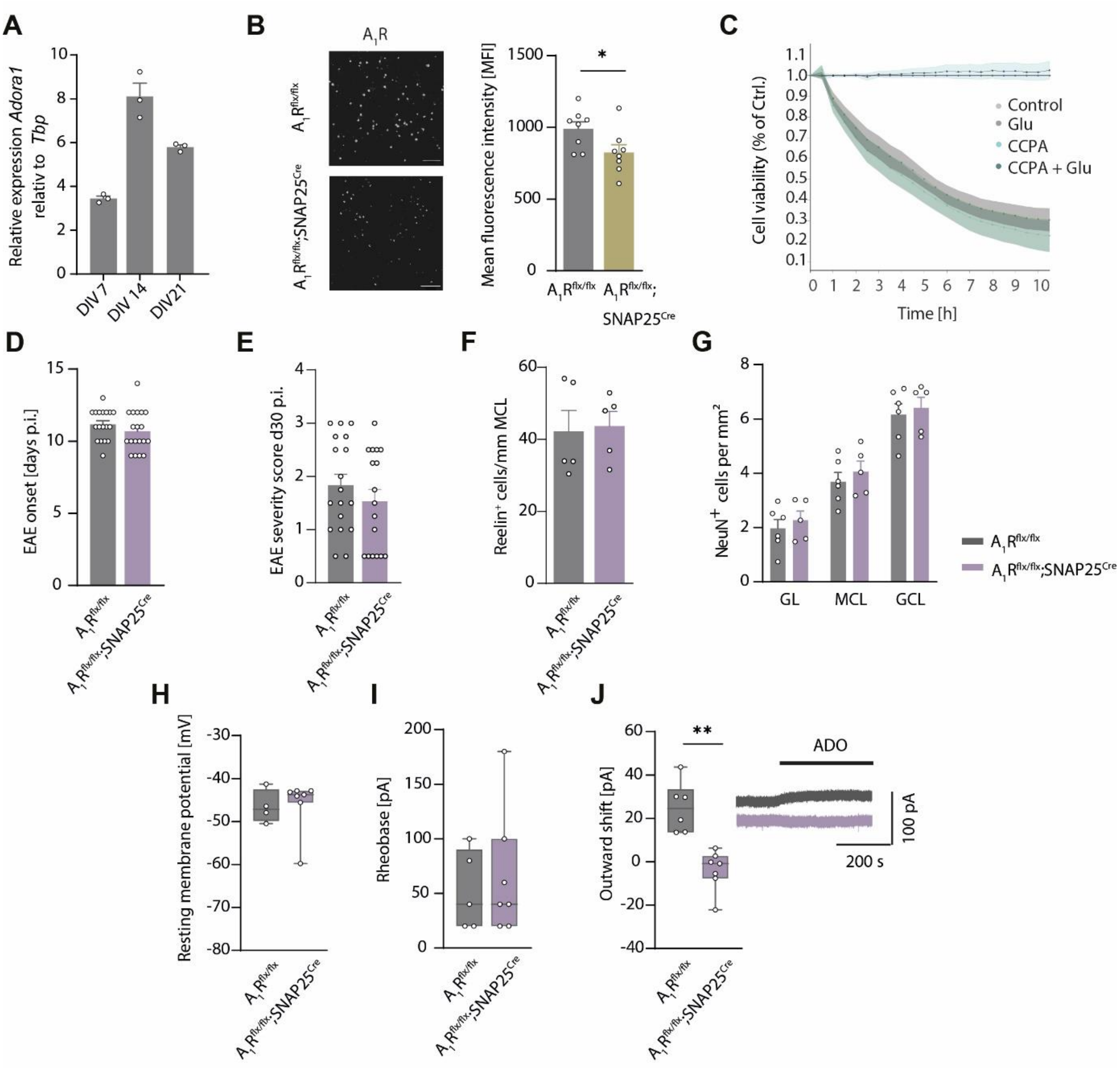

Supplementary Figure 4 | Neuronal A_1R is not involved in glutamate excitotoxicity *in vitro* and neuronal injury in experimental autoimmune encephalomyelitis. (A) Primary neuronal cultures of the mouse olfactory bulb. Relative RNA expression of *Adora1* measured by qRT-PCR at day of days *in vitro* (DIV) 7, 14 and 21. **(B)** Representative image and quantification of mean fluorescence intensity in immunocytochemical staining of A_1R in $A_1R^{flx/flx}$ and $A_1R^{flx/flx}$;SNAP25^{Cre} neuronal cultures (n = 4 biological replicates per group with two technical replicates each). **(C)** Cell viability of wildtype olfactory bulb primary neuronal cultures DIV16-18 over a time course of ten hours after treatment with glutamic acid (5 µM) preincubated with 100 nM 2-chloro-N6-cyclopentyladenosine (CCPA) or medium control compared to the respective controls (n = 4 biological replicates with at least 5 technical replicates each). **(D)** Day of onset in experimental autoimmune encephalomyelitis (EAE) (BF_{10} = 0.307). **(E)** EAE severity score at the final day 30 p.i. in $A_1R^{flx/flx}$ and $A_1R^{flx/flx}$;SNAP25^{Cre} (n = 18 per group, BF_{10} = 0.669). **(F)** Reelin-positive cells in the mitral cell layer based on immunohistochemical staining at peak of EAE day 15 p.i. in $A_1R^{flx/flx}$ and $A_1R^{flx/flx}$ SNAP25^{Cre} (n = 5 per group, BF_{10} = 0.531). **(G)** Quantification of NeuN-positive cells in the glomerular layer (GL, BF_{10} = 0.651), mitral cell layer (MCL, BF_{10} = 0.958) and granular cell layer (GCL, BF_{10} = 0.666) of the olfactory bulb 30 days p.i. (A1Rflx/flx, n = 6; A1Rflx/flx;SNAP25Cre, n = 5).

(H) Resting membrane potential in mitral cells of $A_1R^{flx/flx}$ (*n* = 4) and $A_1R^{flx/flx}$;$SNAP25^{Cre}$ (*n* = 7) (BF_{10} = 0,494). (**I**) Rheobase (first suprathreshold step of current injection) in mitral cells of $A_1R^{flx/flx}$ (*n* = 5) and $A_1R^{flx/flx}$;$SNAP25^{Cre}$ (*n* = 7) (BF_{10} = 0,505). (**J**) Adenosine-induced (100 µM) outward current in mitral cells of $A_1R^{flx/flx}$ (*n* = 6) and $A_1R^{flx/flx}$;$SNAP25^{Cre}$ (*n* = 7) (*P*= 0,003). *P < 0.05, **P < 0,01.
